# Injection Molding and Special Injection Molding Technologies of Polymer and Polymer Composites

**DOI:** 10.3390/polym18010124

**Published:** 2025-12-31

**Authors:** Jian Wang

**Affiliations:** 1 Interdisciplinary Research Center for Intelligent Nanomedicine Translation, Beijing University of Chemical Technology, Beijing 100029, China; jian.wang@mail.buct.edu.cn or wjj_0107@163.com; 2 State Key Laboratory of Organic-Inorganic Composites, Beijing University of Chemical Technology, Beijing 100029, China; 3 College of Mechanical and Electrical Engineering, Beijing University of Chemical Technology, Beijing 100029, China

Injection molding is one of the most widely employed manufacturing processes for the mass production of polymer products, owing to its high efficiency, reproducibility, and versatility [1]. As polymer materials and composite systems continue to evolve, the requirements placed on injection molding have intensified—particularly with respect to dimensional precision, structural integrity, functional integration, and processing efficiency. Modern applications increasingly demand components with complex geometries, tailored mechanical performance, and embedded functionalities such as sensing capabilities or conductivity. Meeting these demands not only requires the precise control of processing parameters, but also a deep understanding of the behavior of materials, mold design, tooling engineering, and process dynamics. Accordingly, the injection molding workflow spans a series of interconnected aspects, including material characterization, structural and product design, mold engineering, equipment optimization, adaptive regulation strategies, and advanced process control techniques [2,3,4].

Parallel to conventional injection molding, numerous specialized or “special” molding technologies have been developed to expand the capabilities of the process. Examples include insert injection molding and overmolding, injection–compression molding (ICM), microcellular and foam injection molding (FIM/MIM), gas- and water-assisted injection molding (GAIM/WAIM), multi-material molding, co-injection, and micro-injection molding (µIM) [5,6,7,8,9]. These technologies broaden the achievable processing window, enabling the formation of multi-functional, lightweight, and structurally complex components that would be challenging or impossible to produce using standard injection molding alone. By improving melt-flow behavior, reducing residual stresses, and controlling microstructural evolution, these methods enhance both the performance of the components and the production yield.

Emerging research is increasingly emphasizing the industrial implementation and optimization of these special molding routes for polymers and polymer composites. Advanced processes such as GAIM, WAIM, microcellular foaming, ICM, co-injection, and micro-/nano-scale precision molding are rapidly maturing [10], offering improved control over dimensional accuracy, internal morphology, surface quality, and functional integration [11]. When combined with high-performance engineering polymers, bio-based materials, nanocomposites, fiber-reinforced systems, and functional fillers, these processes enable the production of lightweight, high-strength, and multifunctional components suitable for automotive, aerospace, electronic, medical, and energy applications [12,13].

Contemporary injection molding research is increasingly driven by a mechanistic understanding of the complex multiphysics phenomena underlying polymer flow, heat transfer, pressure evolution, and phase transformation. The interaction between flow dynamics, thermal histories, pressure fields, and crystallization or foaming significantly influences microstructure and thus determines mechanical, thermal, and functional performance [14,15]. Advances in multiscale simulation, in situ characterization, and data-driven modeling—including machine learning-assisted simulations—are improving predictive capability and strengthening the quantitative links between process settings and final properties [16,17,18]. These insights are essential for defect minimization, rational process design, and stable, high-efficiency manufacturing.

Looking forward, the field is expected to advance along three interrelated dimensions: high-precision processing, intelligent manufacturing, and sustainable production. The integration of advanced sensors, online monitoring systems, and closed-loop process control will allow for real-time regulation and predictive quality management [18]. At the same time, lightweighting strategies, the adoption of bio-based materials, and recycling-oriented processing are anticipated to reduce energy consumption, minimize environmental impact, and improve resource efficiency [19,20,21,22]. The combined application of digital twins (DTs), artificial intelligence (AI)-driven optimization, and multiphysics simulation is likely to further extend the role of special injection molding in advanced manufacturing sectors, including electronics, biomedical devices, and energy-related applications [23,24]. Together, these developments will continue to expand the role of injection molding—and its specialized variants—in next-generation manufacturing sectors.

This Special Issue of *Polymers* is devoted to exploring both the traditional fundamentals and the frontiers of injection molding, especially as they pertain to polymer composites, intelligent control, and novel process variants. More than seventeen submissions were received, and, after rigorous peer review, ten outstanding contributions (nine research articles, one review) were accepted. Together, these papers illuminate new directions in design, process control, and materials for modern injection molding. A brief overview of each contribution is provided below.

## 1. Contribution 1: Injection Molding of Highly Graphite-Filled Polypropylene

Kerling et al. [25] addresses the significant processing challenges inherent in injection molding highly filled thermoplastics, focusing on a polypropylene composite containing 80 wt% graphite for bipolar plate applications. The extreme filler content drastically increases melt viscosity and alters thermal behavior, severely impeding flow and complicating mold filling. Using integrated experimental and numerical analysis, the authors demonstrate that standard Cross-WLF viscosity models fail to predict the flow behavior under high pressure, largely due to pressure-induced crystallization causing premature solidification. The work underscores the necessity of using pressure-dependent viscosity data, obtained via advanced rheometry, to achieve accurate simulations and optimize the manufacturing of these demanding, thin-walled components.

## 2. Contribution 2: Injection-Molded Compliant Constant-Torque Mechanisms

Uyen et al. [26] investigate the design and optimization of injection-molded polymeric compliant constant-torque mechanisms (CTMs), with emphasis on torsional stability under varying load conditions. The study integrates finite element analysis (FEA), experimental torsion tests, and ANN modeling to evaluate the coupled effects of structural geometry and processing parameters on mechanical performance. The results reveal that geometric configuration primarily governs torque–rotation behavior, whereas molding conditions significantly influence performance consistency. The observed discrepancies between the numerical and experimental results highlight the limitations of simplified material models in capturing the nonlinear time-dependent behavior of injection-molded polymers, underscoring the need for refined constitutive representations in the design of functional polymer components.

## 3. Contribution 3: Switchover Strategies in Injection Molding

Bielenberg et al. [27] provide a comprehensive review of the evolution of switchover strategies in injection molding, systematically tracing the shift from operator-dependent empirical practices to data-driven adaptive control frameworks. The authors identify velocity-to-pressure (V–P) switchover as a critical factor affecting dimensional stability, part weight uniformity, and residual stress development. Strategies are categorized into approaches that suppress process-sensitive disturbances and those relying on real-time adaptive control, revealing a trend toward pressure-gradient- and deformation-based monitoring such as tie-bar elongation and mold separation. The review further highlights the increasing relevance of indirect sensing, ML-assisted process control, and predictive modeling, while outlining challenges related to material dependency, mold complexity, and cross-platform transferability.

## 4. Contribution 4: Dual-Network Toughening of Bio-Based PA610 Composites

Zhou et al. [28] examine the design of dual-network architectures to enhance the toughness of bio-based long-chain polyamide 610 (PA610) composites. Rheological analysis and mechanical testing establish a well-defined percolation threshold and quantitatively evaluate interfacial interactions using percolation theory. The synergistic combination of ethylene terpolymer (PTW) and maleic anhydride-grafted styrene–ethylene–butylene–styrene (MAH-g-SEBS) balances toughness and processability, while ultra-high-molecular-weight polytetrafluoroethylene (UHMW-PTFE) promotes semi-interpenetrating network formation. These findings clarify the role of restricted polymer chain mobility in energy dissipation and mechanical reinforcement, providing a mechanistic basis for designing high-performance sustainable polymer composite systems.

## 5. Contribution 5: Enhanced VARTM with Bidirectional Pressure Regulation

Shen et al. [29] conducted a systematic investigation of a modified Vacuum-Assisted Resin Transfer Molding (VARTM) process incorporating bidirectional pressure regulation to optimize resin infusion and consolidation. The study combines theoretical modeling based on Darcy’s law and percolation theory with COMSOL 6.0 simulations and experimental validation, providing a detailed characterization of in-plane, transverse, and three-dimensional resin flow in multilayer fibrous preforms. The results indicate that staged vacuum and external pressure significantly enhance flow-front stability, reduce porosity, and improve fiber volume fraction, leading to superior thickness uniformity and mechanical performance. This work elucidates the coupled effects of pressure fields and resin transport, offering insights into defect suppression in liquid-composite-molding processes.

## 6. Contribution 6: Out-of-Mold Sensing for Thin-Walled Injection Molding

Cheng et al. [30] develop an out-of-mold sensing framework for process optimization and adaptive quality control in hot-runner thin-walled injection molding. By monitoring nozzle pressure and tie-bar strain, the authors construct online quality indicators, including peak pressure, temporal features, viscosity index, and clamping force deviation. An MCU (microcontroller unit)-based adaptive control algorithm compensates for process drift via cycle-to-cycle adjustment. The results demonstrate that stable part quality can be achieved through non-invasive sensing, supporting robust monitoring and control strategies in high-speed manufacturing environments.

## 7. Contribution 7: Weld-Line of Injection Molding of Glass-Fiber-Reinforced PA6

Nguyen et al. [31] investigate weld-line mechanical integrity in glass-fiber-reinforced PA6 composites formed by injection molding. Focusing on PA6 reinforced with 30 wt% glass fibers, the study evaluates the influence of filling time, packing conditions, melt temperature, and mold temperature on tensile strength and elongation at break. A framework integrating mechanical testing, scanning electron microscopy (SEM), and ANN–genetic algorithm (GA) modeling establishes quantitative structure–process–property relationships. The results reveal that weld-line performance exhibits differential sensitivity to processing variables, with packing pressure and melt temperature being dominant factors, providing actionable insight for defect-tolerant component design.

## 8. Contribution 8: Conformal Cooling Channel Design for Injection Mold

Nguyen et al. [32] address tooling-level thermal management via design and optimization of conformal cooling channels (CCCs) to improve temperature uniformity on mold cavity surfaces. By integrating design of experiments (DoE), response surface methodology (RSM), computational fluid dynamics (CFD) simulations, and experimental validation with additively manufactured molds, the authors demonstrate that CCCs enable more homogeneous temperature distributions and improved thermal responsiveness compared to conventional straight-drilled channels, emphasizing the strategic role of integrated tooling design in cycle-time reduction and quality stabilization.

## 9. Contribution 9: Microcellular Injection Molding for In-Mold Fabric Impregnation

He et al. [33] explored a hybrid strategy combining MIM with supercritical nitrogen (SCN) and insert molding for in-mold impregnation of glass fiber fabrics (GFF) using polypropylene (PP). Morphological characterization, mechanical testing, and process simulation revealed that reduced viscosity improves melt flow, fabric impregnation, and cell nucleation. Injection temperature exerts competing effects, enhancing interfacial bonding while potentially affecting foam stability. Observed spatial variations in impregnation quality and mechanical performance reflect the inherent complexity of balancing lightweighting and structural reliability in integrated foaming–impregnation processes.

## 10. Contribution 10: Fabric Insert Injection Molding for Self-Reinforced Composites

Wang et al. [34] investigate a fabric insert injection molding strategy for two-component self-reinforced polyethylene composites based on UHMWPE fabrics and a high-density polyethylene (HDPE) matrix. Experimental optimization and numerical simulation elucidate impregnation behavior and structure–property relationships. By exploiting the processing window arising from the melting temperature difference between UHMWPE and HDPE, improved melt flow and interfacial wetting are achieved without compromising fabric integrity. Simulation-assisted analysis provides detailed insights into local temperature, viscosity, and pressure distributions, supporting scalable routes for recyclable, self-reinforced thermoplastic composite manufacturing.

The contributions collected in this Special Issue highlight the rapid advancements and expanding possibilities of injection molding and special injection molding technologies. They collectively demonstrate how synergistic progress in materials, process design, tooling innovation, and intelligent control is transforming polymer and composite manufacturing. I would like to express my sincere appreciation to all the authors for their excellent contributions, the reviewers for their constructive insights, and the *Polymers* editorial team for their professional support. I anticipate that this Special Issue will stimulate continued innovation and inspire# further research at the intersection of advanced molding technologies, high-performance polymers, and intelligent manufacturing systems.

## Data Availability

Not applicable.
